# Synthesis, spectral characterization, lethal dose (LD_50_) and acute toxicity studies of 1,4-Bis(imidazolylazo)benzene (BIAB)

**DOI:** 10.1016/j.heliyon.2021.e07969

**Published:** 2021-09-09

**Authors:** Hussein Ali Kadhim Kyhoiesh, Mohammed K. Al-Hussainawy, Azal Shakir Waheeb, Khalid J. Al-Adilee

**Affiliations:** a Ministry of Education, Directorate of Education Al-Muthanna, Al-Samawah, AL-Muthanna, Iraq; b Department of Chemistry, College of Science, University of Al-Muthanna, Al-Samawah, Iraq; c Department of Chemistry, College of Education, University of Al-Qadisiyah, Diwaniya 1753, Iraq

**Keywords:** Imidazole, FE-SEM, XRD, Antibacterial, LD_50_, IC_50_

## Abstract

The preparation and spectral identification of new heterocyclic azo ligand 1,4-Bis(imidazolylazo)benzene (BIAB) was prepared by reacting a diazonium chloride salt solution of 1,4-diaminobenzene with imidazole in alkaline ethanolic solution. Differing spectral techniques have been used to study the structure of the azo dye ligand (BIAB) such as Elemental analysis (C.H.N), ^1^H-NMR, Mass spectrum, UV-Vis, FT-IR, XRD, FE-SEM and thermal analysis (TGA-DTA). The pathogenic activities of the synthesized ligand (BIAB) was tested in vitro against the sensitive organisms *Staphylococcus aureus* (Gram-positive) and *Escherichia coli* (gram-negative) as antibacterial and *Aspergillus Niger* and *Candida albicans* as antifungal. The activity data show that the ligand (BIAB) higher antibacterial and slightly antifungus activity in comparison to the standard antibacterial (*Amoxicillin*) and antifungal (*cycloheximide*) drugs. The acute toxicity studies (LD_50_) was calculated using by Miller and Tainter methods (Estimated Probity Units) for the calculation of LD_50_. In this study, different doses (600, 1000, 1300, 1800, 2500 and 3600 μg/ml) of the (BIAB) was administered orally to the different groups of mice. The results exhibited high acute toxicity with LD_50_ of 1020.23 mg/kg upon intraperitoneal administration in mice. The antioxidant properties of the ligand was examined using the DPPH radical scavenging technique. IC_50_ was also determined at 224.17 μg/ml.

## Highlights

• BIAB has been prepared, spectrally characterized and bioactivity assays (antimicrobial, anti-oxidative and LD_50_).
• (BIAB) was tested in-vitro for *Staphylococcus aureus*, *Escherichia coli*, Aspergillus Niger and *Candida albicans*.
• (BIAB) have been shown to have acute toxicity lethal doses (LD_50_) 1020.23 mg/kg.
• The half-maximal inhibitory concentration (IC_50_) is 224.17 μg/ml.

## 1 Introduction

During 1916–1920, G.N. Lewis and I. Langmuir, who proposed that electron transfer would form ionic species, laid the foundations of modern chemical bonding theory. Once the bonds have formed, however, (often called a coordinate bond), they are not distinctive from a 'non-normal covalent bond [1, 2, 3]. It was often proposed that one of such atoms has shared electrons in bonds. In biological systems (for example, heme and chlorophyll), certain essential forms of coordinated compounds occur [4]. The imidazole and derivatives characterized ligands azo dyes of heterocyclic compounds as highly effective against most of the elements of the periodic table as chemistry complexes. Using azo imidazolyl azo in different fields have been used in medicine, science and technology, giving results are of great importance in life [5, 6]. We are now in the process of developing heterocyclic azo mixtures. The central fragment of various natural and biological structures is has been formed by different imidazole derivatives that are an essential class of heterocycles [7]. Due to their strong biological activity, they hold a special role in the field of medical chemistry [8]. One of the most studied organic compounds contains heterocyclic nitrogen, mostly because of its significance in the pharmaceutical [9] and chemical industries [10]. Although inorganic heterocyclic compounds can exist, they usually require one or more elements in the ring structure, such as sulfur, oxygen or nitrogen [11]. As the number of non-carbons substituted by carbon atoms is most generally assumed to be heteroatomic compounds [12]. The aromatic and non-aromatic rings can be used for the structures. The derivatives can be divided into two broad areas as a bunch of heterocyclic materials: aromatic and non-aromatic [13].

The chelation of Ag(I), Cu(II), Zn(II), Cd(II), Hg(II), and Pb(II) with CPLs based on Merrifield resin containing the imidazolylazobenzene (I) and 1,4-bis(imidazolylazo)benzene (II) chelating fragments is of particular interest. The development of five-membered chelate nodes requires the participation of azo-nitrogen and pyridine-nitrogen in the chelation process [14].

A wide range of chemical compounds, including those vital to human survival, such as vitamins and minerals, as well as medications, affect human health. Natural ingredients, by definition, have superior properties in terms of potency and stability when it comes to human wellbeing. Owing to the difficulty of meeting the global demand for natural materials due to their scarcity in nature, it is necessary to manufacture synthetic substances in significant amounts. This may include so-called nature equivalent compounds, which are natural molecules synthesized in an identical or slightly modified molecular structure to boost the molecule's biological function. Heterocycles are one of the most common scaffolds used in medicines and other pharmaceutically related substances. Heterocyclic nuclei's extraordinary capacity to function as biomimetic and reactive pharmacophores has primarily led to their special importance as conventional primary ingredients in a variety of medications. In modern drug research, the use of heterocycles as scaffolds with a high degree of diversity has become a major priority [13, 14, 15, 16].

In our present work, we report the measurements of 1,4-Bis(imidazolylazo)benzene, spectral characterisation and LD_50_ (BIAB). Preparation of the substance (BIAB), studied with various spectral methods, (TGA-DTA), ^1^H-NMR, ^13^C-NMR, Mass, UV-Vis, Infrared FT-IR, XRD, FE-SEM, and C.H. N.

## 2 Materials and methods

### 2.1 Chemicals and solvents

The syntheses were carried out with analytical reagent grade chemicals and solvents, and they were utilized without additional purification. provided fine chemicals such as Imidazole, 1,4-diaminobenzene, Sodium nitrite, 1,1-Diphenyl-2-picrylhydrazyl (DPPH), HCl, NaOH, DMSO, DMF, EtOH, MeOH, Deionized water, Amoxicillin, cycloheximide, MS (mannitol salt) and potato dextrose (PDA) agars were purchased from commercial suppliers (B.H.D (England), Sigma-Aldrich (Germany), Honeywell Fluka (UK), Scharlu (España), and others.

### 2.2 Physical measurements

Elemental microanalyses of ligand (BIAB) was determined using EA 300 (C.H.N.S) Element analyzer. The ^1^H & ^13^C NMR spectra were obtained on a Bruker 400 MHZ spectrometer in DMSO-d_6_ as the solvent, using TMS as the internal reference. Mass spectra of ligand (BIAB) was recorded using a Shimadzu Agilent Technologies 5973C at (70 eV). The electronic spectra were measured on a T80-PG double beam (UV-Vis) spectrophotometer in absolute ethanol using a quartz cuvette of 1 cm path length in the range of 200–1100 nm. FT-IR spectra (KBr disks, 4000–400 cm^−1^) were recorded using a Shimadzu 8400 S. X-ray diffraction (XRD) measurements were performed using a Bestec Aluminium anode-Germany X-ray diffractometer with (Cu Kα) radiation (λ = 1.5418 °A) in the range of (20–80^°^)Θ. Thermal analysis (TGA-DTA) were investigated with a PL-TG instrument from 25 to 900 °C under a nitrogen atmosphere with a heating rate of (10 °*C min*^−1^). Field emission scanning electron microscopy (FE-SEM) images were obtained on a MIRA3 TESCAN. The SMP, Stuart instrument was applied for the recording of the melting point or decomposition temperature of the ligand, capillary tube. The pH measurements were performed with a Philips PW 9421. The compound was created using PerkinElmer ChemBioDraw software and then optimized using PerkinElmer ChemBio3D software.

### 2.3 Synthesis of 1,4-Bis(imidazolylazo)benzene (BIAB)

**Figure 1:**
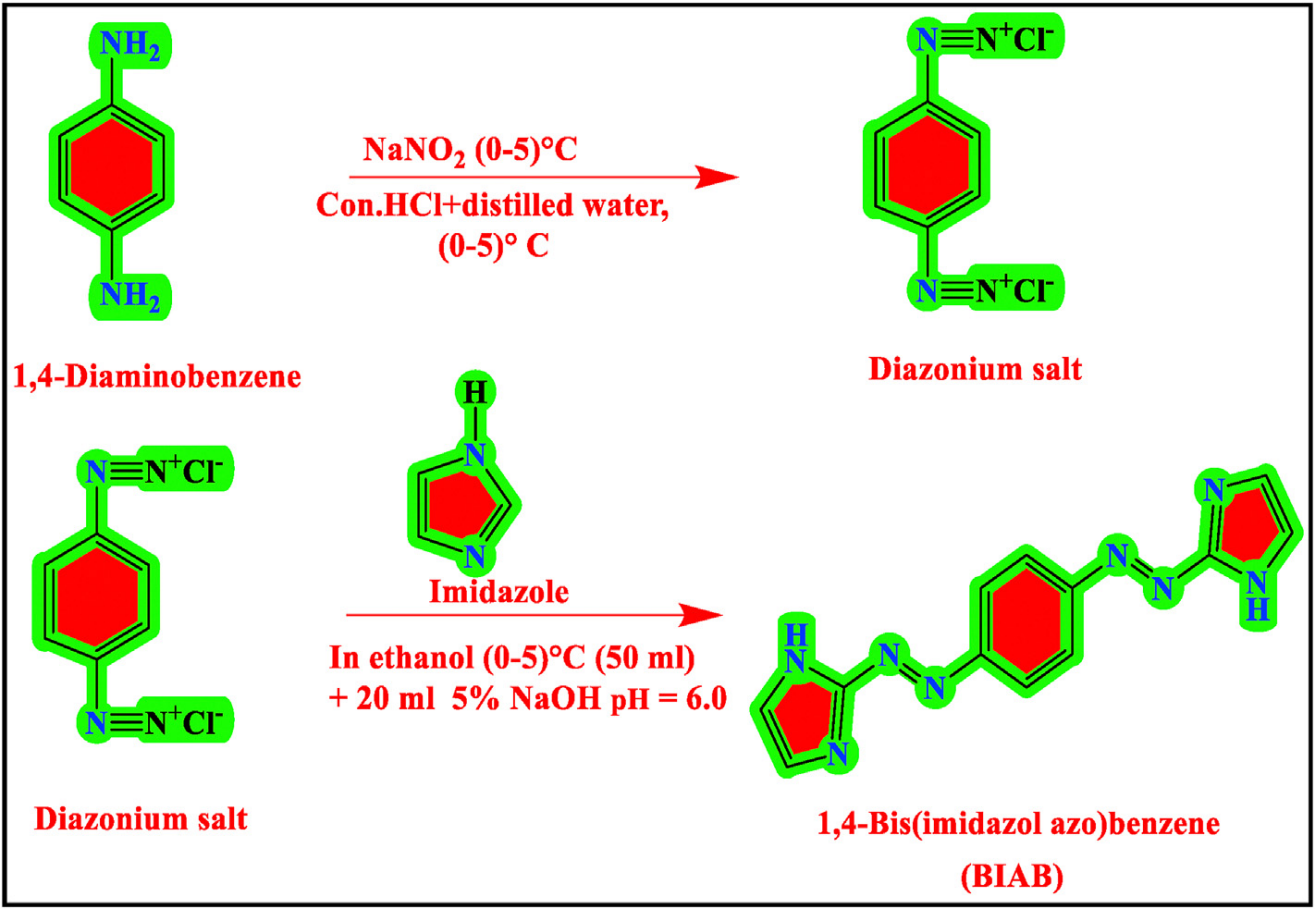
Synthesis of azo ligand 1,4-Bis(imidazolylazo)benzene (BIAB).

The ligand (BIAB) was synthesized by the following methods suggested by Al-Adilee et al. [17, 18] has included the new heterocyclic 1,4-Bis(imidazolylazo)benzene (BIAB) with some revision Figure 1. Dissolving 1.8 g (0.01 mol) of 1,4-diaminobenzene in 25 mL distilled water and 4 mL of concentrated hydrochloric acid (HCl) was used to produce a diazonium solution. A beaker was placed in an ice bath, and the contents of the beaker stirred, cooling up (0–5) °C to the filtered solution. In a combination of 25 ml distilled water a solution of 1.4 g (0.02 mol) of sodium nitrite (NaNO_2_) (0–5) °C was added to the mixture and aged for around 30 min. With cooling and constantly ripening at (0–2)°C the resulting diazonium chloride solution was applied to a 500 mL beaker containing 1.6 g (0.02 mol) of imidazole dissolve in 50 mL of ethanol and 20 ml of 7% sodium hydroxide was ripened for 3 h in an ice-bath and acidification with dilutes of hydrochloric acid pH = 6,0 at (0–5)°C in ice-bath. The raw product was filtered out, distilled water cleaned, purified from ethanol and dried by recrystallization. The obtained 1,4-bis(imidazolylazo)benzene (BIAB) was eventually dried for several hours in the oven at 50 °C and is held in a desiccator by anhydrous dryers. The reaction yield was 70%, and its color was purple crystals and m.p = 229 °C. ^1^H-NMR (400 MHz, DMSO-*d*6) δ 12.24 (s, 2H, N–H), 7.64 (s, 4H, C-H_benzene ring_), 7.11–6.89 (m, 4H, C-H_imidazole ring_), 2.52 (s, solvent DMSO). FT-IR, υ(N–H) 3202cm^−1^, υ(C-H_aromatic_) 3090 cm^−1^, υ(C–H _aliphatic_) 2861 cm^−1^, υ(C=N) 1628 cm^−1^, υ(N=N) 1437 cm^−1^, υ(C=N) 1628 cm^−1^, υ(C–N)_Imidazole_ 1250 cm^−1^ and 794 cm^−1^, υ(C–N=N–C) and υ(C=N–N=C) 1339 cm^−1^, 1317 cm^−1^ and 679 cm^−1^ *m/z*: calculated for C_12_H_10_N8 (266.268). C.H.N.S, X-ray, TGA-DTA, FE-SEM and UV-Visible spectra explained the molecular composition of 1,4-Bis (imidazolylazo) benzene. The following Figure 1 summarizes the 1,4-Bis(imidazolylazo)benzene method of preparation (BIAB).

### 2.4 Microbiological investigation

In this study, two different species of gram-positive and negative bacteria with the sensitivity test system were examined on the inhibitory biological effects of the compounds prepared. The test bacteria is *Staphylococcus aureus* (MTCC 3160) and *Escherichia coli* (MTCC 723). Furthermore, the antifungal activates were tested against (*Aspergillus niger*) (MTCC 1881) and (*Candida albicans*) (MTCC 3958). Nutrient Broth, Mueller Hinton and potato dextrose (PDA) agars were used. The technique (Gram's Method) was also used to diagnose bacteria [19, 20]. Inhibitory zone diameters were measured after 24 h and 7 days, for bacteria and fungi, respectively. The antibacterial and antifungal activities were done at 100 μg/mL concentrations in DMSO solvent by the agar diffusion method. The usual antibacterial and antifungal drugs were *amoxicillin* and *Cycloheximide*, respectively.

### 2.5 Animals

Lab mice (25–30 g) were utilized after being acclimatized in laboratory conditions for 7 days and were typical of similar age (10–12 weeks). The animals had access to food and water, and the sun and dark cycles were shorter than the 12:12 LD cycle. Temperatures of 65–75°F (18–23 °C) with 40–60% humidity.

### 2.6 Acute toxicity studies

The acute toxicity investigations were carried out using the usual approach by Miller and Tainter [21, 22, 23] using the method (Estimation of probity units), in which male albino mice were divided into six groups, each containing ten animals (LD_50_ determination). The ligand dosages were reduced by half in each group $\sqrt{1/2}$ (3600, 2500, 1800, 1300, 1000, and 600 mg/kg body weight). The lethal dose for half of the animals was determined by administering these concentrations orally via an infected tube and recording the indications of poisoning and death within 24 h (LD50).

The animals were kept under constant observation for the first 2 h and then every 4 h after that. There were reports of death and surviving animals after 24 h. The LD50 was calculated both technically and graphically. First, the death percentage was computed using the calculation below, and then the ratio was modified to 0 % and 100 % (Eqs. (1) and (2)) [24, 25];

(1) For 0% death = 100 × (0.25/n)

(2) For 100% death = 100 × [(n- 0.25)/n]

where n is the number of animals in each category.

### 2.7 DPPH radical scavenging assay

The ligand's antioxidant activity (BIAB) is measured by its ability to scavenge the stable free radical 2,2-diphenyl-1-picrylhydrazyl (DPPH). As the color changes from purple to yellow, the stable free radical DPPH displays an electrical absorption band limit at 517 nm. When the odd electron of the DPPH radical is combined with hydrogen from a free radical scavenging antioxidant to form reduced DPPH, the absorption band narrows [26]. A 0.3mM DPPH solution was produced in DMSO and applied to the ligand in 1 mL at different concentrations (0, 25, 50, 100, 200, and 400 μg/mL). The reaction mixture was continuously stirred and kept at 25 °C in the dark for 60 min. At 517nm, the absorbance of the produced reaction mixture was measured, with ascorbic acid as a positive control. This was done with three replications for each concentration. The percentage of DPPH radicals scavenged was determined using the formula below (equation 3) [27];

(3) $$\text{SCV\% ​}=\text{ ​}\left(\frac{{\text{ ​ ​ ​ ​ ​A}}_{\text{control ​}}-{\text{ ​A}}_{\text{sample ​ ​}}}{{\text{A}}_{\text{Control}}}\right)\times 100$$

where A_Control_ is the absorbance of the control reaction (containing all reagents except the test compound) and A_Sample_ is the absorbance of the test compound. The IC_50_ value, known as the concentration of sample required to induce 50% inhibition, was calculated by plotting the percentage of DPPH-scavenging activity against the sample concentration.

## 3 Result and discussion

### 3.1 Characterization of 1,4-Bis(imidazolylazo)benzene (BIAB)

The BIAB, 1,4-Bis(imidazolylazo)benzene, was purple crystals. In certain organic solvents, including ammonia, methanol, ethanol, acetone, DMF and DMSO, ligand (BIAB) is stable and soluble and is soluble in water. The experimental result of the simple study of prepared 1,4-Bis(imidazolylazo) benzene (BIAB) is consistent with the theory. In order to test the purity of the azo dye (BIAB), elemental analysis C.H.N.S is used. Elemental analyses of the bis(imidazolylazo)benzene show that the carbon present within the (BIAB) is 54.06% and hydrogen contain (3.92%). Similarly, the nitrogen content in the (BIAB) is 42.02 %, which is entirely owing to the integrated nitrogenous functional group, according to the elemental analysis of ligand azo.

### 3.2 ^1^H-NMR spectra

**Figure 2:**
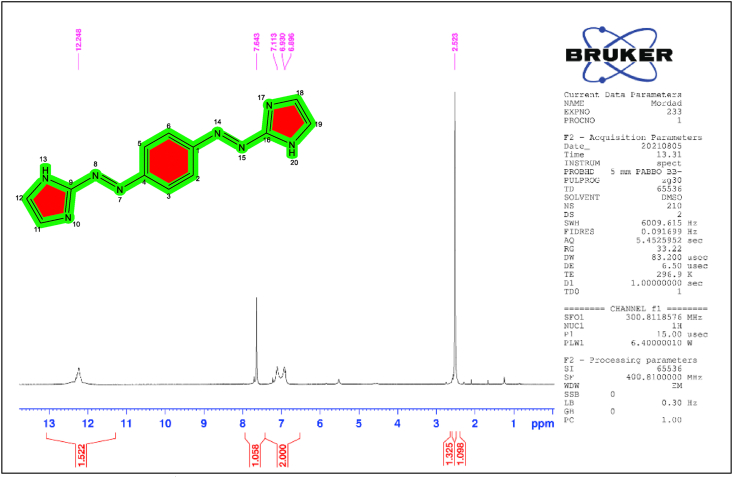
^1^H-NMR spectrum of 1,4-Bis(imidazolylazo)benzene (BIAB).

The ligand 1,4-Bis(imidazolylazo)benzene (BIAB) proton magnetic resonance spectrum (^1^H-NMR) was calculated as a TMS as an internal reference (400 _MHZ_) with DMSO-d^6^ as a solvent and the following peaks were observed in Figure 2. The free ligand spectrum (BIAB) showed the low field signal at δ = 12.24 ppm (s,2H) due to the existence in positions 13 and 20 of the imidazole group (N–H). At the same time, the (BIAB) ligand showed a signal at δ = 7.64 ppm (s,4H of benzene ring) at positions 2,3,5 and 6. A signal at δ = 6.89–7.11 ppm (m, 4H of imidazole ring proton) at positions 11, 12, 18 and 19. Although the signal at δ = 2.52 ppm (s, solvent proton) is due to the influence of DMSO solvent [28].

### 3.3 ^13^C-NMR spectra

**Figure 3:**
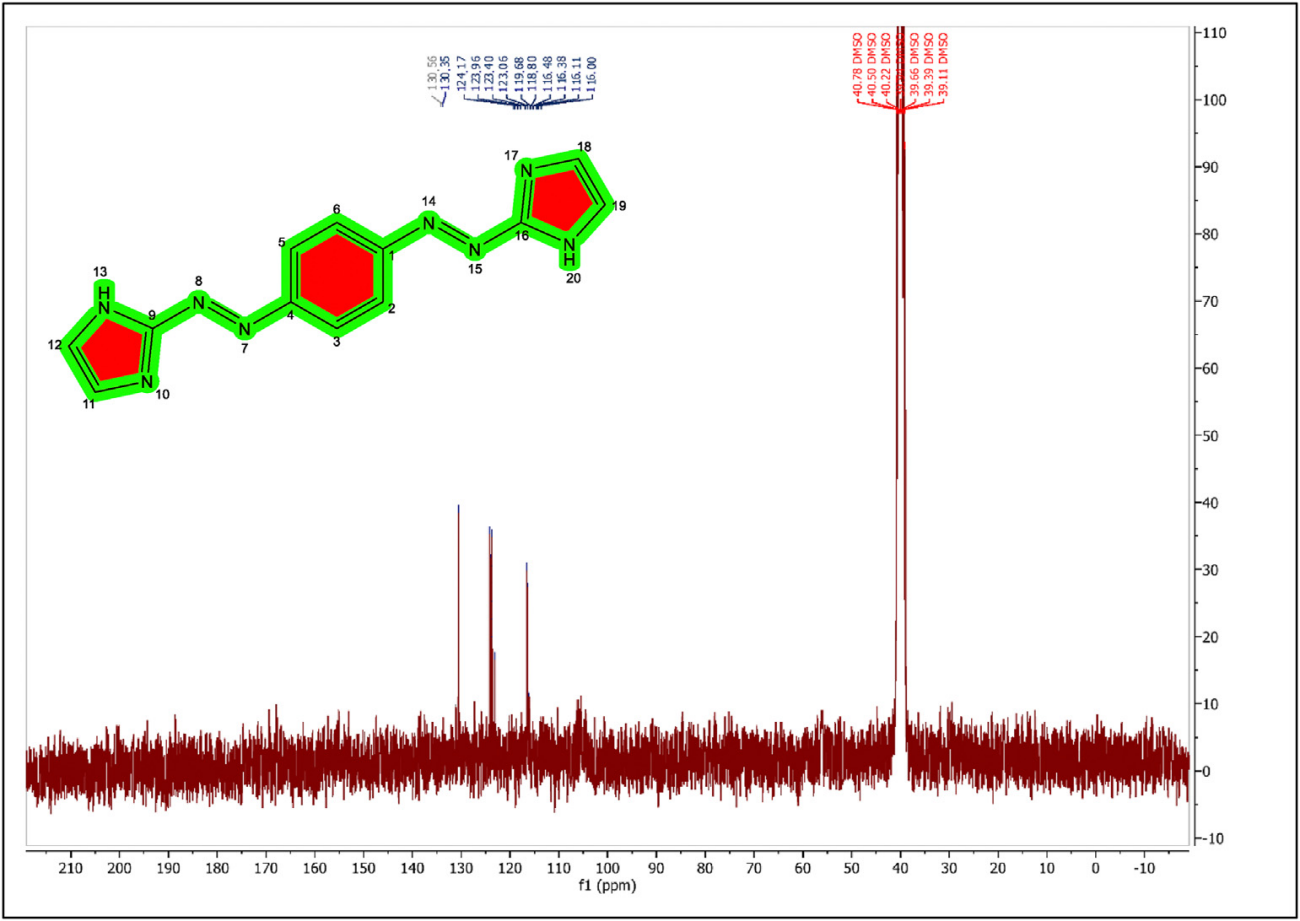
^13^C-NMR spectrum of the ligand (BIAB).

The ^13^C-NMR of the (BIAB) was investigated, and signals were observed due to the presence of various carbon atom types. Table 1 and Figure 3 show the assignment of the ^13^C-NMR spectra of the (BIAB).

**Table 1:** Assignment of the^13^C-NMR spectrum of the ligand (BIAB).

BIAB		
Chemical Shift (ppm)	Position	Associated group
130.56	C_9_	-imidazole-C
130.35	C_16_	-imidazole-C
124.17	C_1_	-Ar-C
123.96	C_4_	-Ar-C
123.40	C_11_	-imidazole-C
123.00	C_12_	-imidazole-C
119.68	C_18_	-imidazole-C
118.80	C_19_	-imidazole-C
116.48	C_7_	-Ar-C
116.38	C_9_	-Ar-C
116.11	C_17_	-Ar-C
116.00	C_18_	-Ar-C
39.11–40.78	-	DMSO-d6

### 3.4 Mass spectral analysis

**Figure 4:**
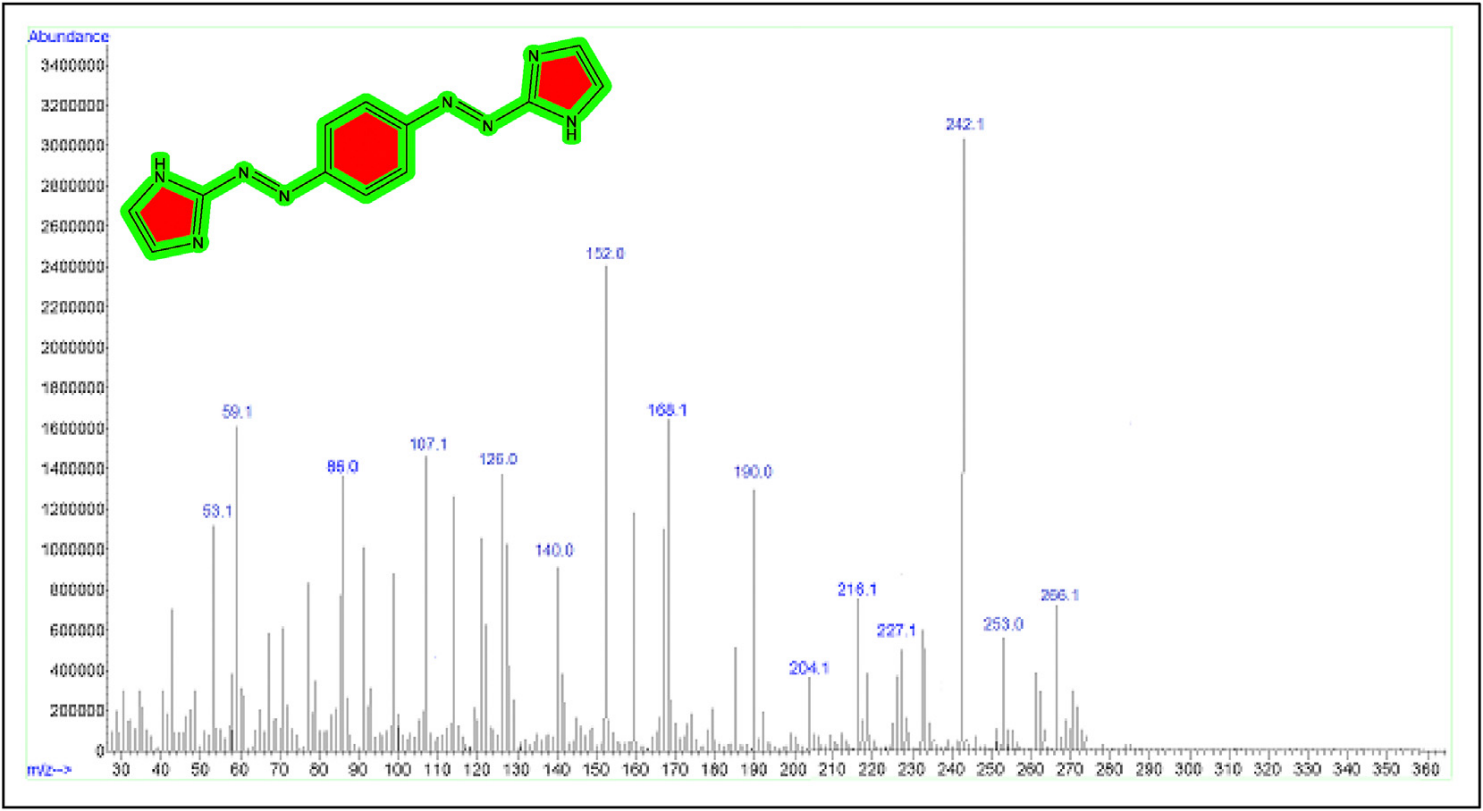
Mass spectrum of ligand (BIAB).

The mass spectrum can be utilized to confirm the ligand's structure as well as its complexes. Multiple peaks were seen in the fragmentation ligand (BIAB) mass spectrum (Figure 4). Table 2 lists all of the mass spectrum data in detail. The mass spectrum of the novel ligand (BIAB) display a base peak at m/z+ = C_12_H_10_N8 (266.1), which is ascribed to the ligand's original molecular weight (BIAB) (266.268), [C_12_H_10_N8] (19.87%). This data matches the chemical formulas well. Figure 4 illustrate the pattern of ligand (BIAB).

**Table 2:** Physical properties and elemental analysis for ligand (BIAB).

Compound	Color	m.p C°	Optimal pH	Yield %	Molecular Formula (M.wt) (g/mol)	Found% (Calc.)			
						C%	H%	%N	%M
BIAB	purple	229	6.5	70	C_12_H_10_N_8_ (266.268)	54.06 (54.13)	3.92 (3.79)	42.02 (42.08)	–

### 3.5 Electronic spectral studies

**Figure 5:**
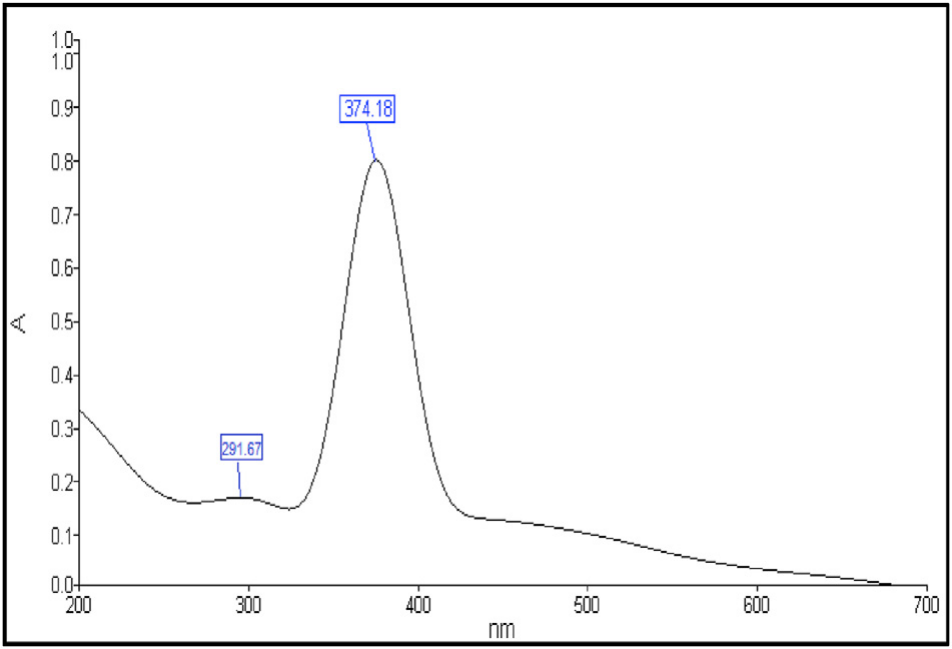
UV-visible spectra of ligand (BIAB).

In the fresh ethanol solution (10^−3^M) at room temperatures, the electronic absorption spectrum of 1,4-bis(imidazolylazo)benzene (BIAB) was reported. Table 3 summarizes the spectral details from the azo dye ligand (BIAB). The free ligand electronic spectrum is distinguished by two U.V-visible absorptions. These bands are 291 nm (34364 cm-1) and 374 nm (26738 cm^−1^). A transition from π →π∗ within the imidazole ring is the primary band. Although n →π∗ transition was due for the second band, the existence of a band with a double bond resulted, Besides hetero atom, an ion-pair electron, such as (=C=N-), in addition to the intermolecular charge transfer in the imidazole ring were also present from the phenyl ring into the azo group (-N=N-) into the imidazole ring [29, 30]. Azo dye ligand (BIAB) UV-visible spectra are seen in Figure 5 and Table 3.

**Table 3:** The spectral data of the azo dye ligand (BIAB).

Compound	λ_max_ (nm)	Absorption bands(cm^−1^)	Transitions
BIAB	374	26738	n →π∗
	291	34364	π →π∗

### 3.6 Infrared spectra

**Figure 6:**
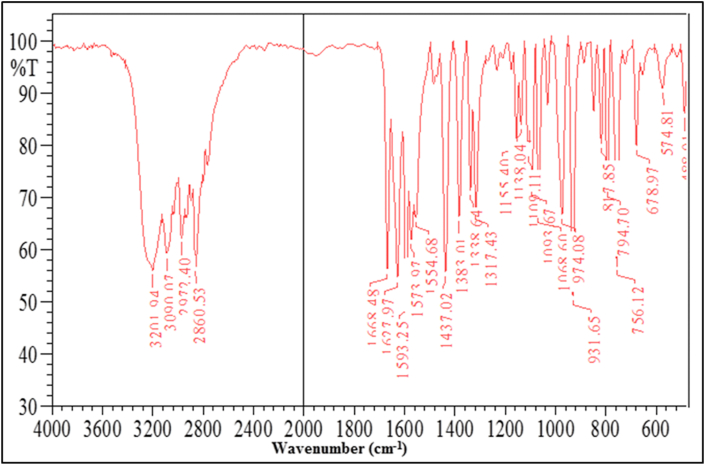
FT-IR Spectrum of ligand (BIAB).

Spectral infrared data (KBr disk) of 1,4 Bis(imidazolylazo)benzene (BIAB) (Table 4). The υ(N–H) imidazole ring has been assigned to a broad 3202cm^−1^ in the FT-IR range of the free bisazo ligand [31, 32]. The band stays in the same free ligand region (BIAB). The ligand (BIAB) spectrum is typical of two strong bands with an aromatic and aliphatic value of 3090 cm^−1^ and 2861 cm^−1^, respectively. The medium band found in the free ligand at 1628 cm^−1^ was credited to υ (C=N) stretch of the azomethine (C=N). The azo group υ (N=N) stretch was attributed to a medium-intensity band at 1437 cm^−1^. The υ (C=N) and υ (C–N)_Imidazole_ stretching vibration appears at 1628 cm^−1^, 1250 cm^−1^ and 794 cm^−1^ respectively. The IR spectra of ligand (BIAB) appear bands at 1339 cm^−1^, 1317 cm^−1^ and 679 cm^−1^ due to υ(C–N=N–C) and υ(C=N–N=C) the presence of diazo group with the conjugated system. The azo dye ligand (BIAB) infra-red spectrum is seen (Figure 6).

**Table 4:** Characteristic FT-IR absorption bands of the ligand (BIAB) in cm^−1^ units (KBr disc).

Group	Ligand
υ (N–H)	3202 Vs.
υ (C–H)_Aromatic_	3090 s.
υ (C–H)_aliphatic_	2861 s.
υ (C=N) and υ (C–N)_Imidazole_	1628 s. 1250 w. 794 w.
υ (N=N)	1437 s.
υ(C=C)	1383 m. 756 w.
υ(C–N=N–C) and υ(C=N–N=C)	1339 m. 1317 m. 679 w.

Vs = very strong, s = strong, m = medium, w = weak, sh = sholder, br = broad.

### 3.7 X-ray diffraction study (XRD)

**Figure 7:**
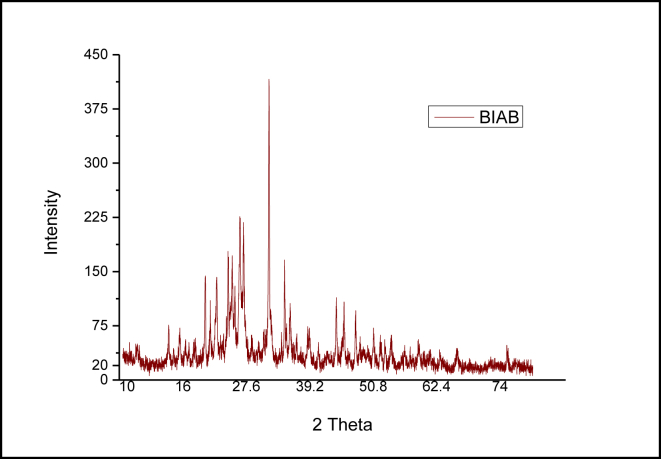
XRD patterns of 1,4-Bis(imidazolylazo)benzene (BIAB).

The intensity of diffracted CuKα radiation was calculated in 2*Ѳ* between 0° and 80° (k = 0.1542 nm; 50 kV and 40 mA). The (XRD) 1,4-Bis (imidazolylazo)benzene (BIAB) patterns appear in this section (Figure 7). A very high degree of crystallinity was shown in the XRD analysis of the prepared (BIAB). Because of the range of high-population x-ray diffraction (BIAB), because of micro-strain and cracking crystalline collapse, as a result of crystalline-scale distortion and domain crystals and domain size distribution [33], it reflects an indicated crystalline nature structure. Bragg's Eq. (4) was used to measure the spacing of reflections [17, 34];

(4) nλ=2dsinθ

where (d) is the spacing between the crystalline levels, (n) is an integer (1,2,3 ...), (λ) is the wavelength of X-ray CuKα = 1.540598 A°, (Ѳ) is the diffraction angle. The d-spacing values and the following data show each peak's 2*Ѳ* values and its relative intensity (Table 5). The findings revealed that the d-spacing of 2.81485 A° suggested the crystalline state of 1,4-Bis (imidazolylazo)benzene (BIAB) with 14 reflections with a maximum of 2*Ѳ* = 31.764°. Crystal sizes (BIAB) have been calculated in the Debye– Scherrer Eq. (5) [30, 35];

(5) D ​=kλ/βCosθ

where (D) is the volume average diameter of the crystallite, (k) is Blank's constant (0.891), (λ) is the X-ray wavelength (0.15405 nm), and (Ѳ) and (β) are the diffraction angle and full width at half maximum of a reported peak, respectively. It was determined the average crystallite size (D) for (BIAB). The following relationship Eq. (6) [36] was well determined for the dislocation density (δ);

(6) $$\text{ ​δ}=1/{\text{D}}^{2}$$

where (δ) is dislocation density, (D) is the average grain size. In Figure 6, the histogram of the particle size distribution (BIAB) has been shown and (Tables 5 and 6).

**Table 5:** Crystallographic data for (BIAB).

Compound	Peak no.	°2Ѳ_(degree)_	d _Spacing__(A°)_	Intensity (I/I_°_)%	FWHM [°2Ѳ]	Crystallite Size D (nm)	Lattice Strain	Dislocation density δ X 10^−4^ (nm)^2^
BIAB	1	20.129	4.40786	32	0.17700	47.63	0.0044	4.407
	2	22.203	4.00050	28	0.23700	35.69	0.0053	7.850
	3	24.314	3.65771	32	0.20050	42.35	0.0041	5.575
	4	25.043	3.55284	29	0.22570	37.68	0.0044	7.042
	5	25.534	3.48573	18	0.14120	60.27	0.0027	2.757
	6	26.448	3.36732	45	0.33030	25.82	0.0061	1.499
	7	27.121	3.28516	41	0.22450	38.04	0.0041	6.910
	8	31.764	2.81485	100	0.17600	49.04	0.0027	4.158
	9	34.613	2.58933	35	0.14620	59.47	0.0020	2.827
	10	35.657	2.51591	18	0.13780	63.28	0.0019	2.497
	11	41.501	2.05345	25	0.15770	52.64	0.0018	3.608
	12	45.475	1.99295	24	0.14480	62.16	0.0015	2.588
	13	47.581	1.90955	18	0.19150	47.37	0.0019	4.456
	14	75.279	1.26135	10	0.17340	60.44	0.0010	2.737

**Table 6:** Crystallographic parameters for (BIAB).

Characteristic	Data and Conditions
Formula	C_12_H_10_N_8_
Crystal colour	Purple
M.wt	266.268
Crystal system	Cubic
Space group	Fm3m
Space group number	225
a (A^°^)	5.6402
b (A^°^)	5.6402
c (A^°^)	5.6402
Alpha (°)	90.0000
Beta (°)	90.0000
Gamma (°)	90.0000
Calculated density (g/cmˆ3)	2.16
Measured density (g/cmˆ3)	2.17
Volume of cell (10ˆ6 pmˆ3)	179.43
Z	4.00
RIR	4.40
Reference code	00-005-0628

### 3.8 Thermal analyses

**Figure 8:**
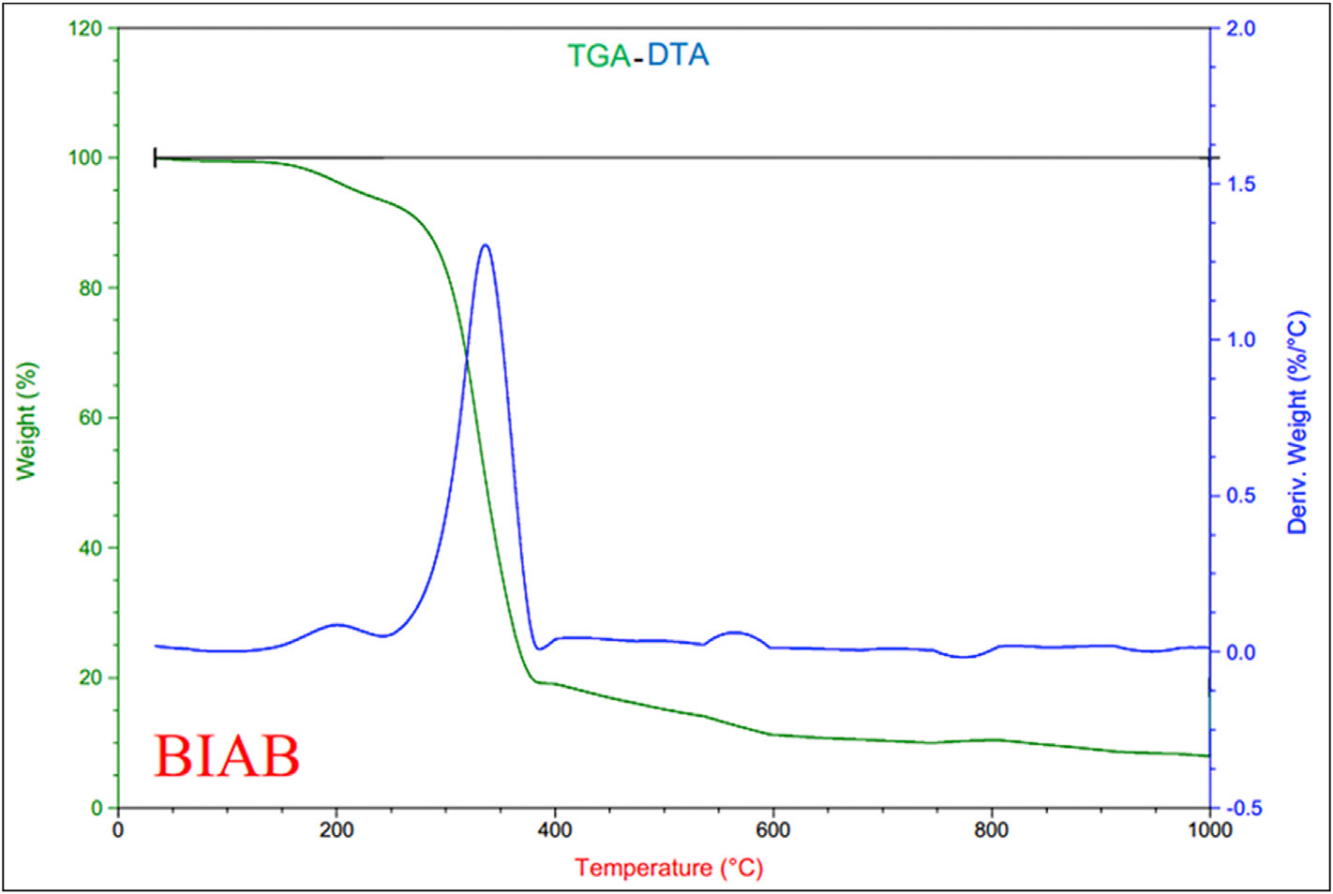
TGA-DTA curves of ligand (BIAB).

Thermogravimetric analysis (TGA) and differential thermal analysis (DTA) was identified with ligand curves (BIAB) (Figure 8). The results of ligand thermogravimetric (BIAB) analyzes are recorded (Table 7). The thermograms were performed in an oxygen atmosphere at 10 °*C min*^−1^ within the range of 22–800 °C. The TGA curve shows three phases of decomposition of the ligand (BIAB) of the formula (C_12_H_10_N_8_). The first step inside the temperature range of 22–243 °C is the moisture and volatile materials, which reflected mass losses of 6.62%. The second decomposition step within the 243–418 °C range corresponds to the loss (C_8_H_5_N_7_) of 74.85 % in these processes. The final decomposition step within the 418–1000 °C range is the loss of further complete fragmentation of a ligand (BIAB), with mass losses of 10.60% at the same time. The peaks of the DTA curve were 200, 335 °C leaving as residue carbon [37, 38].

**Table 7:** Thermoanalytical data (TGA-DTA) of ligand (BIAB).

Compound	TG Range (°C)	Mass loss% Found(Calc.)	Assignment	DTA (°C)	Residue
(BIAB)	22–243	6.62 (6.40)	Evolution of moisture and (C_1_H_5_)	200	_
	243–418	74.85 (74.80)	Loss (C_8_H_5_N_7_)	336	
	418–1000	10.60 (10.59)	Loss of a part of the ligand		

### 3.9 FE-SEM analysis

**Figure 9:**
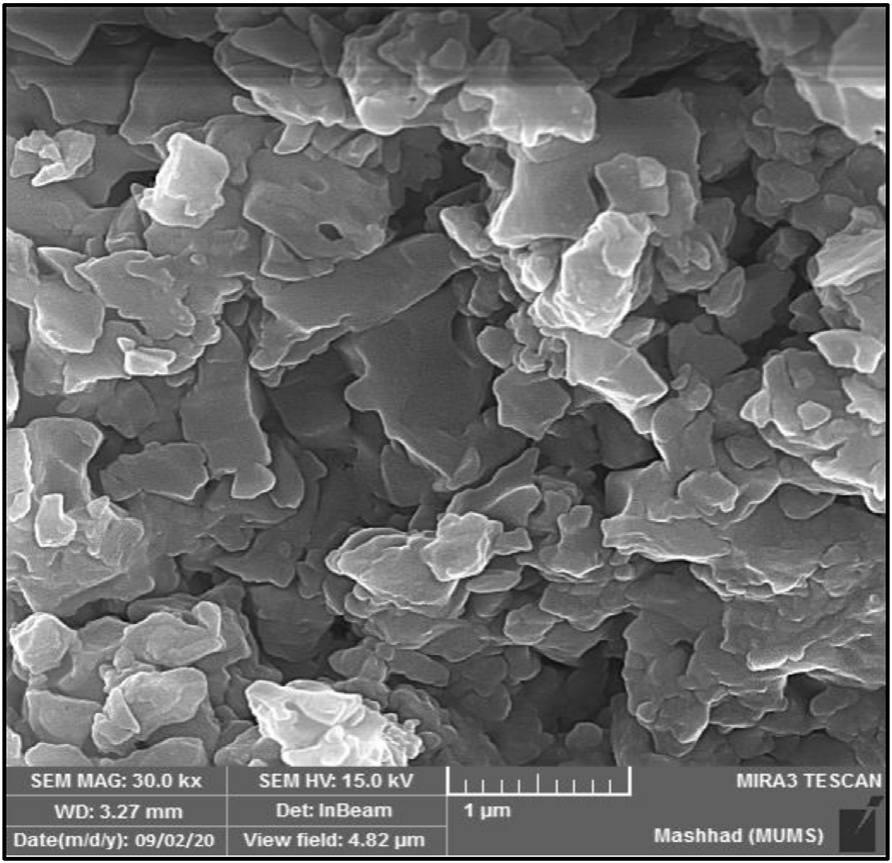
FE-SEM images of ligand (BIAB).

The Field emission scanning electron microscopy (FE-SEM) ligand (BIAB) investigates surface morphology, particulates form, aggregation and propagation of these particles. At 1μm cross-sectional length and a magnification force of Mag = 30.00 KX, a scanning microscope technology was used. The FE-SEM picture was demonstrated in (Figure 9). The ligand (BIAB) shaped peripherally of the spherical shapes at an average size 92 nm with a ratio of less than totality is seen in the FE-SEM image. FE-SEM demonstrated that in some cases, the particles became agglomerated and non-uniform [39]. Nanoparticles with different sizes and morphology have been synthesized in various conditions e.g. isolated particle (100–150 nm), aggregate particles (600–700 nm), irregular multilateral shapes (1.5–2 μm), porous structure (40 nm), and nanoparticles (28–32 nm) [40]. Controlling shape, particle size, and size distribution, as well as developing novel functionalization processes, are all important elements in effectively implementing nanoparticles in various applications [41]. Thermal decomposition techniques, for example, provide an alternative to the regularly used co-precipitation approach for producing nanoparticles with a regulated form, adjustable size, and limited size distribution, giving them distinctive features [42]. The pyrolysis preparation technique and reaction conditions must be modified or revised in order to create consistent nanosized materials. For example, atomizing the precursor solution with ultrasonic waves [43], dispersing the precursor solution with a stable matrix such as zeolite [44] or glass, slowing the reaction rate to obtain nanoparticle films, i.e., the oxide superconducting films formed in vacuum, authorizing the reaction to occur in an inert solvent or inert gas, and using up decomposable polymers or molecules [45]. These changes effectively lower the nanoparticles' crucial formation temperature. They also prevent aggregation and agglomeration of the nanoparticles [46, 47].

### 3.10 Antimicrobial activity

**Figure 10:**
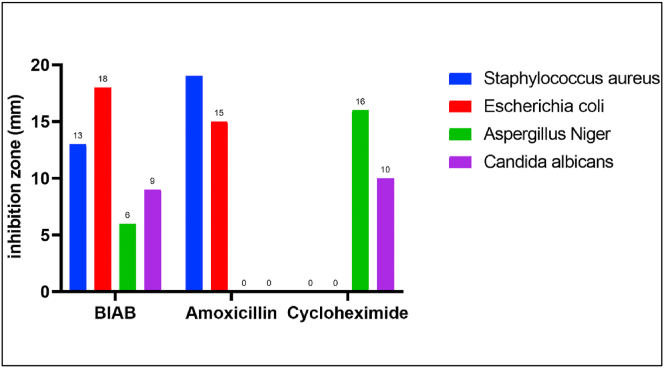
Antimicrobial Activity of ligand (BIAB).

The ligand (BIAB) was examined in vitro against bacteria gram-positive (*Staphylococcus aureus)*, gram-negative *(Escherichia coli),* antifungal *(Aspergillus niger*) and (*Candida albicans)* at the concentration 100 μg/ml shown in Table 8 and Figure 10. Ligand (BIAB) showed direct antibacterial and antifungal activities in comparison to the standard antibacterial (*Amoxicillin*) and antifungal (*cycloheximide*) drugs. Data revealed that 1,4-bis (imidazolylazo)benzene (BIAB) is more effective relative to other bacteria and fungi in *Escherichia coli* strains. The *Escherichia coli* bacteria displayed a high susceptibility to the ligand. The findings showed that the form of compensated compound groups, for example, the group (-N=N- and N–H), in the studied ligand [48, 49, 50], influence the degree of efficacy and the bacteria type influencing them. Fungi, on the other hand, indicated that the majority of the compound tested had moderate to weak active ingredients (inhibition zones ranged from 6 to 9 mm) [51].

**Table 8:** Antimicrobial activity data (zone of inhibition in mm) of azo dye ligand (BIAB).

Compound	Bacteria		Fungi	
	Gram Positive	Gram Negative		
BIAB	*Staphylococcus aureus*	*Escherichia coli*	Aspergillus Niger	*Candida albicans*
	13 mm +++	18 mm +++	6 mm +	9 mm ++
*Amoxicillin*	19 +++	15 +++	---	---
*Cycloheximide*	---	---	16 +++	10 ++

Highly active = + + + (inhibition zone >12 mm).

Moderately active = + + (inhibition zone 9–12 mm).

Slightly active = + (inhibition zone 6–9 mm).

Inactive = - (inhibition zone <6 mm).

### 3.11 Acute toxicity of (BIAB)

**Figure 11:**
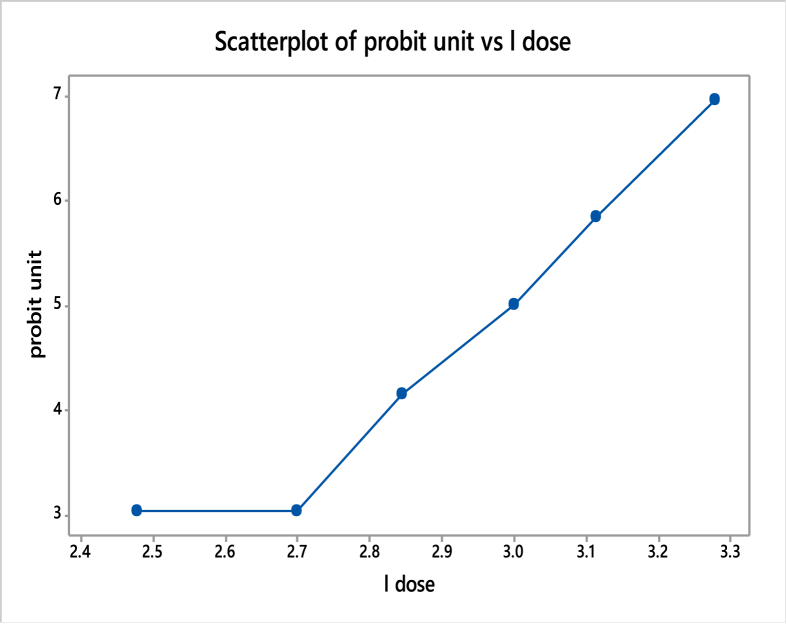
The dose-response graph in terms of probability units.

**Figure 12:**
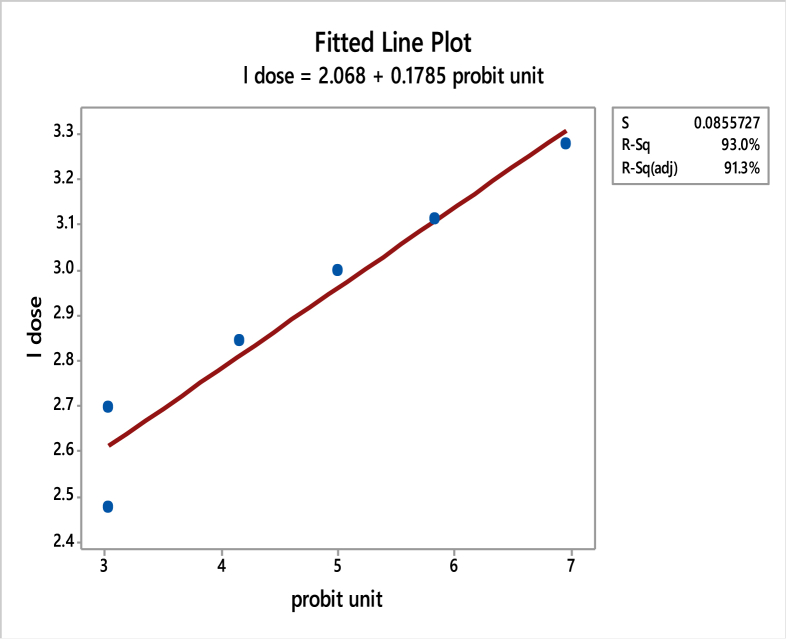
The straight line equation to describe the response.

The results for acute toxicity studies (LD_50_ determination) are tabulated as shown in Tables 9, 10, 11 and Figures 11, 12. The toxicity determinations were carried out in which % mortality observed was 0, 0, 20, 50, 80 and 100%, respectively, for the doses corresponding to 600, 1000, 1300, 1800, 2500 and 3600 mg/kg, respectively. When a plot of log dose (on X-axis) vs. % mortality (on Y-axis) was plotted, a line was obtained, which at probit 5 extrapolated to a value of 3.00087 on X-axis, which in turn corresponds to the value of 1020.23. Thus the LD_50_ of ligand BIAB was found to be 1020.23 mg/kg of the body weight. The drug is practically non-toxic at oral doses, supporting the fact that it was widely used in ancient ages [52, 53, 54, 55, 56]. Toxicity was observed with BIAB the death of all (100%) animals at 3600 mg/kg b.wt.

**Table 9:** Miller & Tainter method of estimation of LD50 [43].

%	0	1	2	3	4	5	6	7	8	9
0	-	2.67	2.95	3.12	3.25	3.36	3.45	3.52	3.59	3.66
10	3.72	3.77	3.82	3.87	3.92	3.96	4.01	4.05	4.08	4.12
20	4.16	4.19	4.23	4.26	4.29	4.33	4.36	4.39	4.42	4.45
30	4.48	4.50	4.53	4.56	4.59	4.61	4.64	4.67	4.69	4.72
40	4.75	4.77	4.80	4.82	4.85	4.87	4.90	4.92	4.95	4.97
50	5.00	5.03	5.05	5.08	5.10	5.13	5.15	5.18	5.20	5.23
60	5.25	5.28	5.31	5.33	5.36	5.39	5.41	5.44	5.47	5.50
70	5.52	5.55	5.58	5.61	5.64	5.67	5.71	5.74	5.77	5.81
80	5.84	5.88	5.92	5.95	5.99	6.04	6.08	6.13	6.18	6.23
90	6.28	6.34	6.41	6.48	6.55	6.64	6.75	6.88	7.05	7.33

**Table 10:** Transformation table for the dose to probits.

(Probits)	(Corrected %)	(%dead)	(Md)	Log dose	(dose) (mg/kg)	(n)	Group
3.04	2.5	0	0	2.477	600	10	1
3.04	2.5	0	0	2.698	1000	10	2
4.16	20	20	2	2.845	1300	10	3
5.00	50	50	5	3.000	1800	10	4
5.84	80	80	8	3.113	2500	10	5
6.96	97.5	100	10	3.278	3600	10	6

Log Dose = 3.00087.

LD_50_ = 1020.23 mg/kg.

**Table 11:** Fitted Line: l dose versus probit unit.

Regression Analysis: l dose versus probit unit					
The regression equation is					
l dose = 2.068 + 0.1785 probit unit					
S = 0.0855727 R-Sq = 93.0% R-Sq(adj) = 91.3%					
Analysis of Variance					

Source	DF	SS	MS	F	P

Regression	1	0.391700	0.391700	53.49	0.002
Error	4	0.029291	0.007323		
Total	5	0.420991

### 3.12 DPPH radical scavenging assay

**Figure 13:**
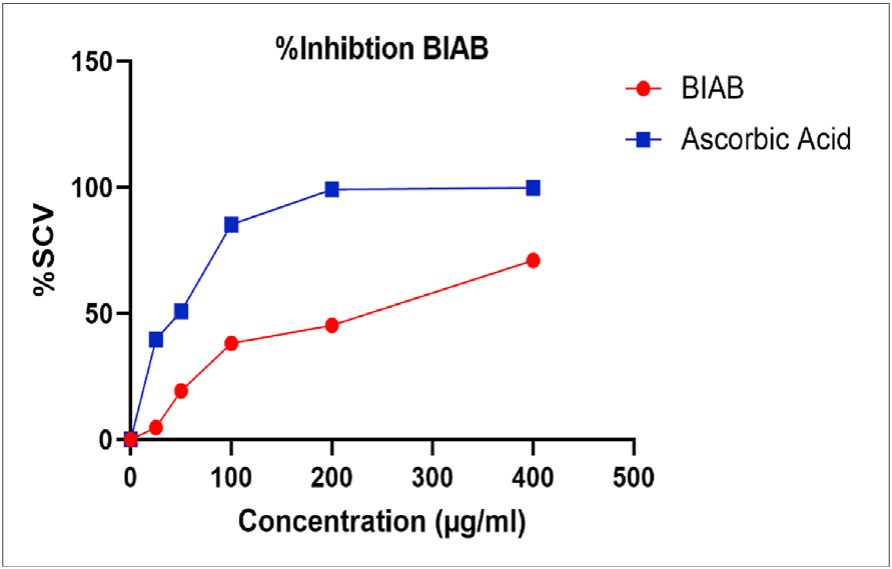
Antioxidant activity of BIAB.

**Figure 14:**
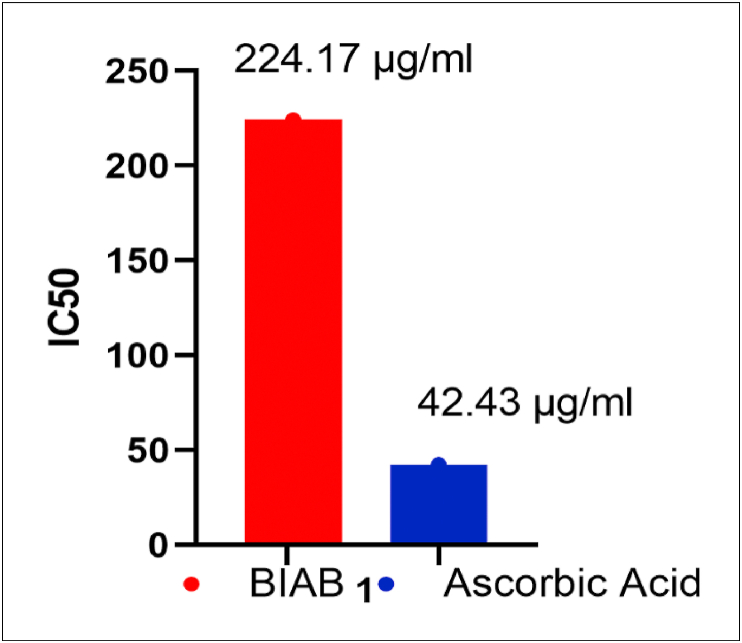
IC_50_ for BIAB and Ascorbic acid.

The DPPH assay is commonly used to determine the ability of a substance to scavenge free radicals and is calculated in terms of IC_50_ values. In the visible field, DPPH has a high absorbance at 517 nm, which will be reduced when the ligand is added (BIAB). The azo group in the tested BIAB can be donated to the DPPH free radical [57]. When an odd electron on the DPPH radical is paired off in the presence of BIAB, it is reduced, and the colour of the solution varies from purple to yellow. The decrease in the absorbance value of DPPH at 517 nm can be used to calculate the BIAB's free radical scavenging function [58]. With increasing BIAB concentrations, scavenging activity increases. At 400 μg/ml, BIAB has a higher antioxidant activity. The findings (Table 12) and (Figures 13,14) show that BIAB outperforms standard ascorbic acid.

**Table 12:** Results of antioxidant activity evaluation for BIAB and Ascorbic acid.

Compound No.	Concentration (μg/ml)					IC_50_ (μg/ml)
	25	50	100	200	400	
	% Antioxidant activity					
Ascorbic Acid	39.65	50.79	85.32	99.21	99.77	42.43
BIAB	4.76	19.34	38.21	45.37	71.11	224.17

## 4 Conclusions

Azo dye ligand 1,4-Bis(imidazolylazo)benzene (BIAB) derived from imidazole was prepared and structurally characterized. The ligand structure and interpretation results (C.H.N), FT-IR, ^1^H-NMR and electronic spectra confirmed this formation. Azo dye ligand has many morphologies, as seen in XRD and FE-SEM, TGA-DTA of ligand (BIAB), as well as a good thermal stabilization result. The biological activity and the extraordinary biological activity of the synthesized BIAB ligand. The analysis indicates that the ligand is highly biological in effectively checked against *Escherichia coli, Staphylococcus aureus, Aspergillus Niger* and the *Candida albicans*. The activity data indicate that the highly active ligand is prepared against all testing species of bacteria. The lethal dose for half of the animal number (LD_50_) of the organic compound (BIAB) was 1020.23 mg/kg, of the animal's weight. As for the half-maximal inhibitory concentration (IC_50_) attain 224.17 μg/ml by examining free radicals.

## Declarations

### Author contribution statement

All authors listed have significantly contributed to the development and the writing of this article.

### Funding statement

This research did not receive any specific grant from funding agencies in the public, commercial, or not-for-profit sectors.

### Data availability statement

Data included in article/supplementary material/referenced in article.

### Declaration of interests statement

The authors declare no conflict of interest.

### Additional information

No additional information is available for this paper.
